# Longitudinal Trends in the Incidence of Hyperactive Delirium and Its Causes of Change After Surgery for Degenerative Lumbar Disease: A Population‐Based Study of 7250 Surgical Patients Over 11 Years

**DOI:** 10.1111/os.14301

**Published:** 2024-12-08

**Authors:** Bang‐Lin Xie, Li‐Zhong Nie, Biao Zhong, Jun Xiong, Miao Nie, Qiu‐Xiao Ai, Dong Yang

**Affiliations:** ^1^ Orthopedic Hospital, The First Affiliated Hospital, Jiangxi Medical College Nanchang University Nanchang China; ^2^ The Key Laboratory of Spine and Spinal Cord Disease of Jiangxi Province Nanchang China; ^3^ Department of Orthopedics Nanchang People's Hospital (The Third Hospital of Nanchang) Nanchang China

**Keywords:** lumbar degenerative disease, postoperative analgesia, postoperative delirium, risk factors, trend of incidence rate

## Abstract

**Objectives:**

Delirium is one of the common complications in elderly patients after spinal surgery. Severe delirium can lead to a series of adverse consequences such as drainage tube removal, wound rupture, patient falls, and severe adverse effects. The current research on POD is mostly small sample studies. This study aimed to investigate longitudinal changes in the incidence of hyper‐postoperative delirium in patients with lumbar degenerative disease at our institution over the past 11 years and to identify the potential causes of these changes.

**Methods:**

This is a retrospective cohort study included 7250 patients who underwent surgical treatment for lumbar degenerative diseases at a single center from 2011 to 2021. These patients were diagnosed with delirium through the Confusion Assessment Method and then diagnosed with high activity delirium through the Richmond Agitation‐Sedation Scale, totaling 130 cases. According to the incidence rate of hyper‐postoperative delirium within 11 years, the trend test is divided into three groups: S1 upward trend (2011–2014), S2 downward trend (2015–2016) and S3 upward trend (2016–2021). The study collected variables from patients before, during, and after surgery, including gender, age, laboratory tests, anesthesia risk score, New York Heart Association cardiac function grading, number of surgical segments, surgical time, estimated intraoperative blood loss, anesthesia medication, and supplementary analgesic medication in the ward within 3 days after surgery. Kruskal Wallis one‐way ANOVA test, Kruskal–Wallis *H* test, or chi‐square test are used to evaluate inter group differences. *p* < 0.05 is considered statistically significant.

**Results:**

The pooled incidence of hyper‐postoperative delirium over the past 11 years was 1.79% (130/7250). The average age of 7250 patients was 54.5 ± 13 years, with a male/female ratio close to 1:1. We statistically analyzed the relevant influencing factors before, during, and after surgery of S1 and S3 in the incidence rate increase group and found no statistical difference between the two groups. Our research results show that the incidence of high activity delirium is correlated with age, number of surgical segments, surgical duration, use of dexmedetomidine, remifentanil, and benzodiazepines, with *p* < 0.05.

**Conclusions:**

The reduced use of dexmedetomidine, increased use of benzodiazepines, and prolonged surgical time are the reasons for the increased incidence of hyper‐postoperative delirium. The joint management of orthopedic surgeons and anesthesiologists during the perioperative period is of great significance in reducing the incidence of hyper‐postoperative delirium in patients undergoing lumbar spine surgery.

## Graphical abstract

Total Incidence trends of hyper‐POD after degenerative lumbar disease surgery from 2011 to 2021. Age and gender subgroups Incidence trends of hyper‐POD after degenerative lumbar disease surgery from 2011 to 2021.

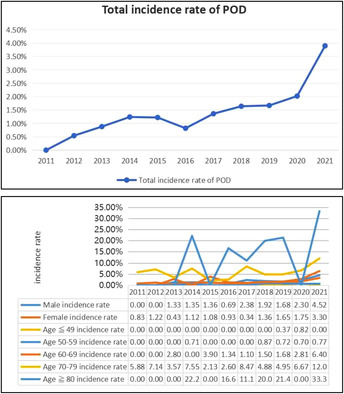

**Abbreviations**
ASA: American Society of Anesthesiologists
BMI: body mass index
CAM: confusion assessment method
CCI: Charlson comorbidity index
EBL: estimated blood loss
hyper‐POD: hyperactive postoperative delirium
ICU: intensive care unit
NYHA: New York Heart Association
PCIA: patient‐controlled intravenous analgesia
POD: postoperative delirium
RASS: Richmond Agitation‐Sedation Scale

## 1 Introduction

Delirium is defined as an acute disorder of attention and cognition with obvious fluctuations. Postoperative delirium (POD) refers to delirium that occurs within 1 week after surgery and has obvious temporal characteristics, mainly within 1–3 days after surgery [1]. Among elderly patients, this is a common, serious but preventable postoperative complication [2]. POD can lead to an increased incidence of serious adverse consequences, such as prolonged hospital stay in the short term, cognitive dysfunction, 30‐day readmission rate, and increased mortality within 1 year [2, 3, 4].

POD episodes can be divided into three subtypes: hyperactive, hypoactive, and mixed [5]. The main symptoms in patients with hyperactive POD (hyper‐POD) are obvious restlessness, fear, nonsense, sudden attacks, hallucinations, and euphoria after surgery. Hyper‐POD accounts for 25% of all POD cases; however, because it is highly destructive, inappropriate or unsafe behaviors of severely agitated patients will bring safety risks (such as falls, extubation, incision dehiscence, and infection), which interfere with the progress of clinical work and are often the most recorded [6]. Although the other two types of delirium account for 75% of POD cases, they are often overlooked by doctors and nurses due to difficulties in identification [7, 8].

POD is often diagnosed using daily mental state tests and validated diagnostic algorithms [9]. POD is common in patients undergoing cardiac surgery, with an incidence ranging from 11.3% to 51.6% [10]. Its risk factors include advanced age, low education level, lifestyle (long‐term smoking, heavy alcohol consumption, etc.), multiple comorbidities, abnormal perioperative laboratory results, intraoperative‐related factors, and environmental conditions [11, 12, 13, 14, 15].

Some studies have reported the incidence (2.8%–8.4%) [2, 16] and risk factors of delirium after lumbar fusion surgery, but most were limited to a short time range and small sample size. This study retrospectively evaluated the dynamic trend of the incidence of hyper‐POD in degenerative lumbar diseases over the past 11 years, conducted a long‐term and systematic analysis of the underlying causes of these changes with the aims: (i) Analyzed the longitudinal trend of its incidence and the underlying causes of the changes;(ii) Exploring how targeted strengthening of perioperative management can effectively reduce the incidence of delirium in spinal surgery.

## 2 Materials and Methods

### 2.1 Clinical Data Research Participants

The study participants included patients who underwent elective surgery under general anesthesia for lumbar degenerative diseases at the First Affiliated Hospital of Nanchang University from January 1, 2011 to December 31, 2021, and screened patients who developed hyper‐POD. This study was approved by the Ethics Committee of the First Affiliated Hospital of Nanchang University (Ethical approval No. [2022] YYL‐K [4‐013]) and complies with all regulations.

### 2.2 Inclusion Criteria for Surgical Patients

The study enrolled patients (1) diagnosed with lumbar degenerative disease (LD), including lumbar disc herniation, lumbar spondylolisthesis, and lumbar spinal stenosis; (2) who underwent surgery under general anesthesia, and (3) underwent elective surgery.

### 2.3 Inclusion Criteria for Hyper‐POD Patients

Hyper‐POD was diagnosed when (1) the patient was evaluated as positive for delirium using the confusion assessment method (CAM) [17] and (2) the Richmond Agitation‐Sedation Scale (RASS) [18] score was > 0.

### 2.4 Exclusion Criteria

The exclusion criteria were (1) delirium developed preoperatively, (2) dementia or other mental illnesses, (3) alcohol consumption or withdrawal, and (4) postoperative acute cerebrovascular disease.

### 2.5 Research Methods

#### 2.5.1 Identification, Diagnosis, and Treatment Process of Hyper‐POD Patients

(1) The patient presented with clinical symptoms after surgery, including irritability, sudden aggression, hallucinations, and gibberish. (2) Preliminary evaluations and documentation were conducted by spinal surgeons and nurses. (3) Psychosomatic doctors were invited to evaluate and diagnose the patient. (4) Corresponding intervention measures were provided (protective restraint, use of sedatives such as oral olanzapine tablets, and intramuscular injection of haloperidol).

#### 2.5.2 Data Collection

Medical records of POD patients were collected, including the following aspects: The demographic data and preoperative variables included sex, age, date of admission, body mass index (BMI = weight (kg)/height^2^ (m)), education level, long‐term smoking history, alcohol abuse history, comorbidities (heart, lung, liver, nervous system, etc.), Charlson comorbidity index (CCI), anesthesia risk score (ASA), NYHA heart function grade, and preoperative laboratory test results (hemoglobin, creatinine, albumin, serum sodium, and serum potassium). Intraoperative variables included the number of surgical segments, anesthesia time, surgery time, estimated intraoperative blood loss (EBL), transfusion status (autologous blood recovery/allogeneic blood), total infusion volume, and anesthesia medication. Postoperative variables included patient‐controlled intravenous analgesia (PCIA) medication use, ICU admission after surgery, laboratory examination on the first day after surgery, duration of closed drainage tube placement after surgery, and supplemental analgesic medication in the ward within 3 days after surgery. This study was approved by the Ethics Committee of the First Affiliated Hospital of Nanchang University (approval no. 20220712).

### 2.6 Statistical Analysis

A trend test was conducted on the incidence of hyper‐POD at our center over the past 11 years, and IBM SPSS Statistics 26 (IBM, USA) was used for statistical analysis. Categorical variables were reported as frequencies and percentages. Normally distributed data were reported as mean and standard deviation (SD), whereas skewed distribution data were reported as median and interquartile range. A linear‐by‐linear association test was used to evaluate the time trend of the hyper‐POD incidence rate. The Shapiro–Wilk normality test was used to examine the distribution of continuous variables. According to the type and distribution of variables, the Kruskal–Wallis one‐way ANOVA test, Kruskal–Wallis *H*‐test, or chi‐square test were used to examine the differences between groups. The above test methods were adjusted for *p*‐values using the Bonferroni correction method, and *p* < 0.05 was considered statistically significant.

## 3 Results

### 3.1 General Information of Hyper‐POD Patients

**FIGURE 1:**
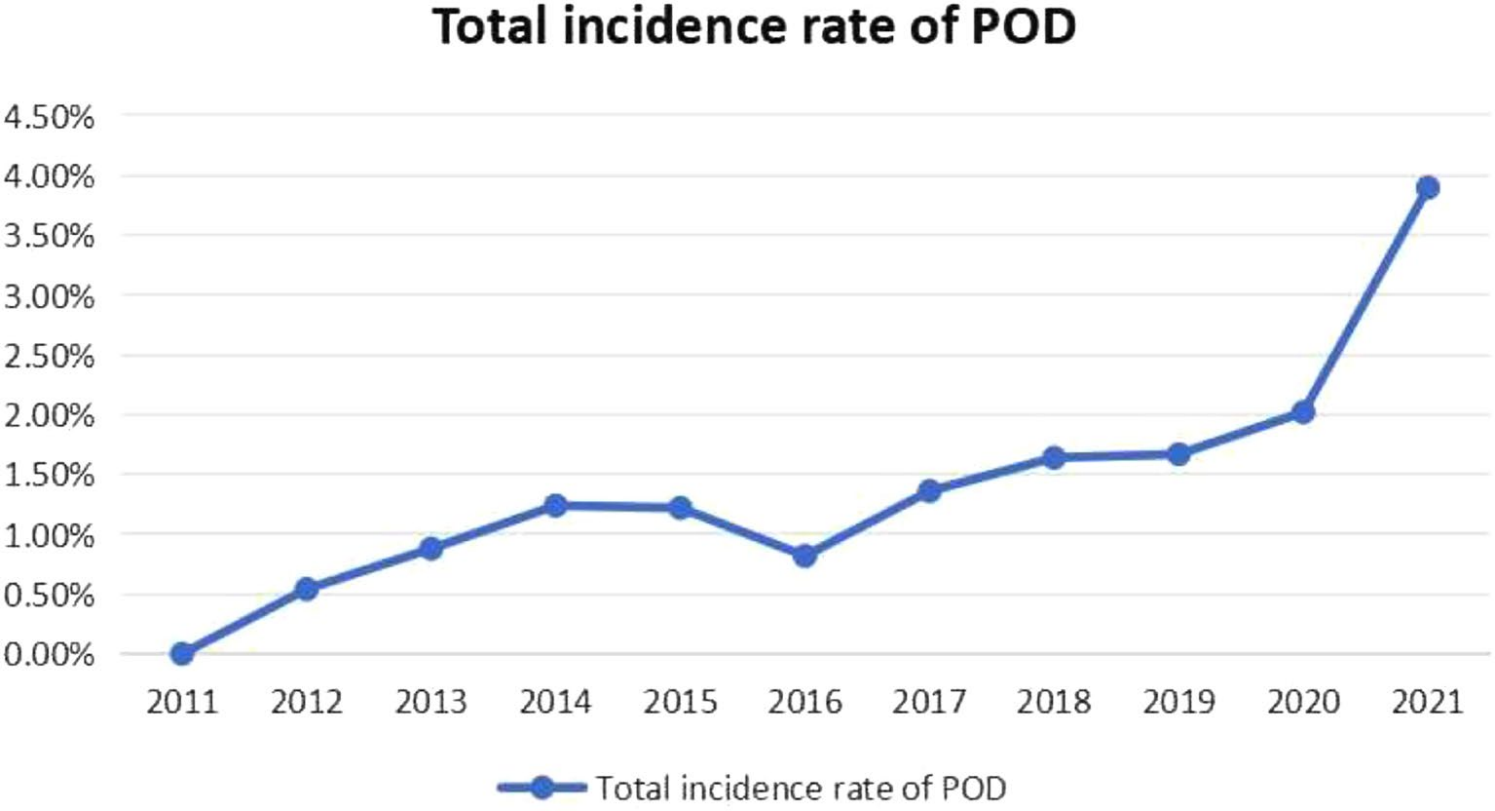
Total Incidence trends of hyper‐POD after degenerative lumbar disease surgery from 2011 to 2021. Time trends in the incidence of hyper‐POD in degenerative lumbar spine disease were assessed using a linear‐by‐linear association test (*p* < 0.001).

**FIGURE 2:**
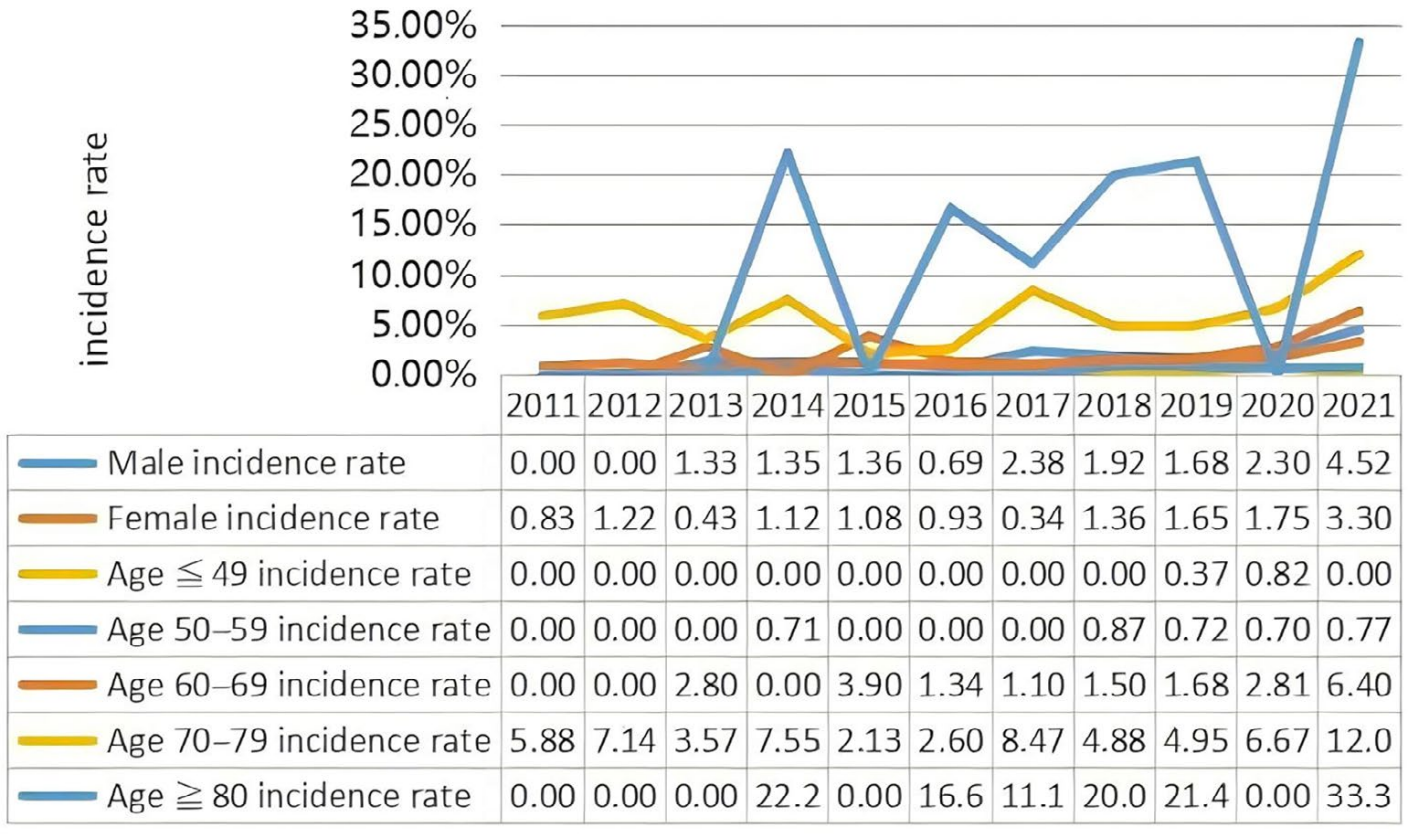
Age and gender subgroups Incidence trends of hyper‐POD after degenerative lumbar disease surgery from 2011 to 2021.

From January 1, 2011 to December 31, 2021, the study included a total of 7250 patients with lumbar degenerative diseases who underwent elective surgical treatment under general anesthesia. Among them, 130 patients developed hyper‐POD, with a comorbidity rate of 1.79%. As given in Table 1, the annual incidence of hyper‐POD in lumbar degenerative diseases has increased from 0.39% in the past 11 years. A trend test was conducted on the incidence of hyper‐POD in our center over the past 11 years. The overall incidence rate of 11 years was trend tested, and the overall incidence rate showed an upward trend, assessed using a linear‐by‐linear association test (*p* < 0.001). From 2011 to 2014, it showed an upward trend; from 2015 to 2016, it showed a downward trend; and from 2017 to 2021, it showed an upward trend. So the timeline was divided into three periods: T1 (2011–2014), T2 (2015–2016), and T3 (2016–2021) (Figure 1). The 7250 surgical patients were divided into three groups for comparison according to the time periods, namely T1, T2, and T3 groups. A total of 130 patients who developed HPOD within the 7250 patients in the three time periods were further divided into three groups: S1, S2, and S3 groups. The average age was 54.5 ± 13 years, with a male/female ratio of nearly 1:1. The age group with the highest proportion was ≤ 49 years old (32.8%), followed by the 50–59 years old group (29.8%) and the 60–69 years old group (25.8%) (Figure 2).

**TABLE 1:** Correlation analysis of factors affecting the overall incidence of hyper‐POD.

Variable	*X* ^2^	*p*
Gender	0.314	0.576
Age	5.841	0.045
Smoking history	0.532	0.466
Cultural level	0.131	0.717
History of drinking	0.197	0.658
Surgical segment	5.096	0.024
Operative time	0.786	0.038
Dexmedetomidine	11.841	0.001
Remifentanil	39.001	0.000
Benzodiazepines	6.368	0.012

### 3.2 Factors Influencing the Incidence of Hyper‐POD

Our research results show that the incidence of hyper‐POD is correlated with age, number of surgical segments, surgical duration, use of dexmedetomidine, remifentanil, and benzodiazepines, with *p* < 0.05 (Table 1). We conducted a statistical analysis of the preoperative, intraoperative, and postoperative influencing factors of the ascending groups S1 and S3 (Tables 2, 3, 4, 5, 6), and found no statistically significant differences between the two groups. Compared with descending group S2, we found that the use of dexmedetomidine, benzodiazepines, and surgical duration affected the incidence of hyper‐POD, with statistical differences (*p* < 0.05) (Table 7). Our results show that the reduction in surgical time is related to the increasing working age and surgical volume of surgeons, as well as the improvement of surgical techniques. At the same time, it is also related to the widespread application of new equipment such as ultrasonic bone knives introduced by hospitals.

**TABLE 2:** Comparison of age, sex, and age group of the total population in the three groups.

	Total (*n* = 7250)	Group T1 (*n* = 1646)	Group T2 (*n* = 1185)	Group T3 (*n* = 4419)	*F/X* ^2^	*p*
Age^a^	54.5 ± 13	52.2 ± 12.8*^,^ **	54.8 ± 12.1*	55.2 ± 13.2**	34.2 (*F*)	< 0.001
Male (*n*, %)^b^	3657 (50.4%)	865 (52.6%)	583 (49.2%)	2209 (50.0%)	4.0 (*X*)	0.134
Age group (*n*, %)^b^						
≤ 49	2378 (32.8%)	702 (42.6%)*^,^ **	391 (33.0%)*^,^ ***	1285 (29.1%)**^,^ ***	100.2 (*X*)	< 0.001
50–59	2164 (29.8%)	452 (27.5%)	358 (30.2%)	1354 (30.6%)	5.9 (*X*)	0.052
60–69	1869 (25.8%)	353 (21.4%)*^,^ **	304 (25.7%)*	1212 (27.4%)**	22.4 (*X*)	< 0.001
70–79	745 (10.3%)	126 (7.7%)*^,^ **	123 (10.4%)*	496 (11.2%)**	16.6 (*X*)	< 0.001
≥ 80	94 (1.3%)	13 (0.8%)*	9 (0.8%)**	72 (1.6%)*^,^ **	9.8 (*X*)	0.007

*Note*: *, **, and ***, statistically significant difference between two groups with the same sign, *p* < 0.05. The *p* values were adjusted using the Bonferroni correction method.

^a^ Kruskal–Wallis one‐way ANOVA.

^b^ *Z*‐test.

**TABLE 3:** Comparison of the incidence of hyper‐POD in the three groups by age.

	Total (*n* = 7250)	Group T1 (*n* = 1646)	Group T2 (*n* = 1185)	Group T3 (*n* = 4419)	*X* ^2^	*p*
Total (*n*, %)^a^	130 (1.79%)	14 (0.85%)**	12 (1.01%)*	104 (2.35%)*^,^ **	14.2	< 0.001
Age group (*n*, %)^b^						
≤ 49	3 (0.12%)	0 (0)	0 (0)	3 (0.23%)	1.5	0.741
50–59	10 (0.46%)	1 (0.12%)	0 (0)	9 (0.66%)	2.5	0.304
60–69	49 (2.62%)	3 (0.85%)	8 (2.63%)	38 (3.14%)	5.6	0.064
70–79	50 (6.71%)	8 (6.35%)	3 (2.44%)	39 (7.86%)	4.7	0.084
≥ 80	18 (19.15%)	2 (15.38%)	1 (11.11%)	15 (20.83%)	0.4	0.907

*Note*: * and **, statistically significant difference between two groups with the same sign, *p* < 0.05. The *p* values were adjusted using the Bonferroni correction method.

^a^ Kruskal–Wallis one‐way ANOVA.

^b^ *Z*‐test.

**TABLE 4:** Comparison of demographic data and preoperative variables among the three hyper‐POD population groups.

	Group S1 (*n* = 14)	Group S3 (*n* = 104)	*H*/*X* ^2^	*p*
BMI (kg/m^2^)^a^	23.3 (17.4, 24.7)	22.9 (20.5, 25.4)	0.2 (*H*)	0.639
Education level (*n*, %)^b^				
Junior high school and below	13 (92.9%)	87 (83.7%)	0.3 (*X*)	0.615
Long‐term smoking (*n*, %)^b^	2 (14.3%)	14 (13.5%)	0 (*X*)	0.933
History of alcoholism (*n*, %)^b^	2 (14.3%)	9 (8.7%)	0.4 (*X*)	0.618
CCI (*n*, %)^b^			2.3 (*X*)	
≥ 3	1 (7.1%)	24 (23.1%)		0.198
ASA (*n*, %)^b^			14.6 (*X*)	
Grade III^c^	7 (50.0%)*	95 (91.3%)*		< 0.001

*Note*: *, statistically significant difference between two groups with the same sign, *p* < 0.05.

Abbreviations: ASA, American Society of Anesthesiologists Classification score; CCI, Charlson Comorbidity Index.

^a^ Wilcoxon test.

^b^ *χ* ^2^ test.

^c^ *t*‐test.

**TABLE 5:** Comparison of intraoperative variables in the three hyper‐POD groups.

	Group S1 (*n* = 14)	Group S3 (*n* = 104)	*X* ^2^/*H*	*p*
Operative segments (*n*, %)^a^				
Level 1	12 (85.7%)*	46 (44.2%)*	8.5 (*X*)	0.004
Level 2	2 (14.3%)	40 (38.5%)	3.2 (*X*)	0.134
≥ Level 3	0 (0)	18 (17.3%)	2.9 (*X*)	0.124
Duration of operation (min)^b^	191 (131.8, 211.3)	180 (140, 230)	721 (*H*)	0.956
EBL (mL)^b^	400 (250, 550)	300 (250, 550)	697.5 (*H*)	0.801
Narcotics				
Dexmedetomidine (*n*, %)^a^	7 (50.0%)	61 (58.7%)	0.4 (*X*)	0.575
Dosages (μg)^b^	25 (20, 37.5)*	30 (30, 40)*	117.5 (*H*)	0.043
Benzodiazepines (*n*, %)^b^	8 (57.1%)	37 (35.6%)	2.4 (*X*)	0.147
Remifentanil (*n*, %)^b^	9 (64.3%)*	95 (91.3%)*	8.6 (*X*)	0.012
Dosages (mg)^b^	1 (1, 1)	1 (1, 2)	297.5 (*H*)	0.113
Sufentanil (*n*, %)^b^	14 (100%)	103 (99%)	0.3 (*X*)	1.000
Dosages (μg)^b^	200 (117.5, 200) *	30 (25, 100)*	130.5 (*H*)	< 0.001
Penehyclidine (*n*, %)^b^	14 (100%)	85 (83.1%)	3.0 (*X*)	0.122
Propofol (mg)^b^	1140 (1010, 1195)	1010 (780, 1170)	639 (*H*)	0.464

*Note*: *, statistically significant difference between two groups with the same sign, *p* < 0.05.

Abbreviation: EBL, estimated blood loss.

^a^ *χ* ^2^ test.

^b^ Wilcoxon test.

**TABLE 6:** Comparison of PCIA medication and postoperative variables among the three hyper‐POD groups.

	Group S1 (*n* = 14)	Group S3 (*n* = 104)	*T*/*X* ^2^	*p*
Analgesic situation^a^				
Unused (*n*, %)^b^	1 (7.1%)	2 (1.9%)	1.4 (*X*)	0.086
Central class (*n*, %)^b^	9 (64.3%)	86 (82.7%)	2.3 (*X*)	0.145
Dezocine (mg)^c^	24.9 ± 19.0*	48.9 ± 21.7*	−3.5 (*T*)	0.001
Other (*n*, %)^b^	4 (28.6%)	16 (15.4%)	1.5 (*X*)	0.253
PCIA				
Pentazocine (*n*, %)^b^	3 (21.4%)*	61 (58.7%)*	19.7 (*X*)	< 0.001
Admission to the ICU (*n*, %)^b^	0 (0)	3 (2.9%)	0.4 (*X*)	1.000
Duration of closed drainage (d)^c^	2.3 ± 0.6*	2.9 ± 1.1*	−2.1 (*T*)	0.040

*Note*: *, statistically significant difference between two groups with the same sign, *p* < 0.05.

Abbreviations: ICU, intensive care unit; PCIA, patient controlled intravenous analgesia.

^a^ Use of analgesics within 3 days postoperatively, which include PCIA and supplemental analgesics.

^b^ *t*‐test.

^c^ *χ* ^2^ test.

**TABLE 7:** Comparison of variables among the three hyper‐POD population groups.

	Total (*n* = 130)	Group S1 (*n* = 14)	Group S2 (*n* = 12)	Group S3 (*n* = 104)	*H*/*X* ^2^	*p*
BMI (kg/m^2^)^a^	22.9 (20.3, 24.7)	23.3 (17.4, 24.7)	22.1 (20.4, 23.4)	22.9 (20.5, 25.4)	0.9 (*H*)	0.639
Education level (*n*, %)^b^						
Junior high school and below	110 (84.6%)	13 (92.9%)	10 (93.3%)	87 (83.7%)	0.7 (*X*)	0.815
Long‐term smoking (*n*, %)^b^	18 (13.8%)	2 (14.3%)	2 (16.7%)	14 (13.5%)	0.4 (*X*)	0.898
History of alcoholism (*n*, %)^b^	11 (8.5%)	2 (14.3%)	0 (0)	9 (8.7%)	1.4 (*X*)	0.511
CCI (*n*, %)^b^						
≥ 3	25 (19.2%)	1 (7.1%)	0 (0%)	24 (23.1%)	4.7 (*X*)	0.088
Operative segments (*n*, %)^b^						
Level 2	49 (37.7%)	2 (14.3%)	7 (58.3%)	40 (38.5%)	5.4 (*X*)	0.063
≥ Level 3	18 (13.8)	0 (0)	0 (0)	18 (17.3)	4.4 (*X*)	0.079
Duration of operation (min)^a^	181 (143.8, 222.5)	191 (131.8, 211.3)	200 (160, 200)	180 (140, 230)	0.1 (*H*)	0.045
EBL (mL)^b^	400 (250, 500)	400 (250, 550)	600 (400, 600)	300 (250, 550)	0.1 (*H*)	0.969
Narcotics						
Dexmedetomidine (*n*, %)^b^	80 (61.5%)	7 (50.0%)*	12 (100%)*, **	61 (58.7%)**	10.0 (*X*)	0.007
Benzodiazepines (*n*, %)^b^	45 (34.6%)	8 (57.1%)*	0 (0)*, **	37 (35.6%)**	10.5 (*X*)	0.004
Sufentanil (*n*, %)^b^	129 (99.2%)	14 (100%)	12 (100%)	103 (99%)	1.6 (*X*)	1.000
Propofol (mg)^a^	1035 (848.8, 1185)	1140 (1010, 1195)	1100 (1080, 1100)	1010 (780, 1170)	0.6 (*H*)	0.741
Analgesic situation^b^						
Unused (*n*, %)^b^	4 (3.1%)	1 (7.1%)	1 (8.3%)	2 (1.9%)	3.5 (*X*)	0.178
Central class (*n*, %)^b^	105 (80.8%)	9 (64.3%)	10 (83.3%)	86 (82.7%)	2.7 (*X*)	0.264
Other (*n*, %)^b^	21 (16.2%)	4 (28.6%)	1 (8.3%)	16 (15.4%)	2.1 (*X*)	0.402
Admission to the ICU (*n*, %)^b^	4 (3.1%)	0 (0)	1 (8.3%)	3 (2.9%)	1.7 (*X*)	0.371

*Note*: * and **, statistically significant difference between the two groups with the same sign, *p* < 0.05. The *p*‐values were adjusted using the Bonferroni correction method.

^a^ Kruskal–Wallis *H*‐test.

^b^ *Z*‐test.

### 3.3 Complications of Hyper‐POD Patients

In total, 23 patients with postoperative high activity delirium developed complications, including 11 cases of drainage tube dislodgement, 8 cases of incision dehiscence, 2 cases of incision infection, and 2 cases of falls.

## 4 Discussion

### 4.1 The Overall Trend of Hyper‐POD Incidence Is Increasing

Our research results show that the overall incidence of hyper‐POD is on the rise. The incidence of hper‐POD is correlated with age, number of surgical segments, surgical duration, use of dexmedetomidine, remifentanil, and benzodiazepines. The results show that the average age of T1, T2, and T3 gradually increases, and the proportion of elderly patients aged ≥ 60 years significantly increases. Therefore, the research team believes that the changes in the incidence of hyper‐POD during this period may be related to the increasing age of surgical patients. A large number of studies have also reported that advanced age is an important risk factor for POD occurrence [19], which is consistent with the results of this study. The research results also showed that with the development of time, the number of lumbar multisegment surgeries (≥ 3 segments) increased, and the number of patients with various comorbidities increased. Therefore, we believe that the gradual changes in the population structure of surgical patients, with increasing age and difficulty of surgery, may be partially responsible for the overall upward trend in delirium incidence.

### 4.2 Opioid Drugs Affect the Incidence of Hyper‐POD

Our research findings suggest that the use of remifentanil can influence the incidence of hyper‐POD. The decrease in the incidence of delirium during the T2 stage might be associated with alterations in the dosage of remifentanil administered during surgery. Numerous studies have demonstrated that the utilization of opioid drugs is one of the triggering factors for delirium [20]. Drug‐induced delirium is hypothesized to result from the overactivity of the dopaminergic system and the underactivity of the cholinergic system [21]. However, not all opioid drugs increase the incidence of delirium. Studies have indicated that the use of pethidine and tramadol elevates the risk of delirium, whereas the use of drugs like morphine has a protective effect [22]. There is no convincing evidence that the risk of delirium in elderly patients depends on the type of opioid [23]. Reducing the use of opioid drugs to alleviate delirium may augment postoperative pain in patients, and pain itself is also a factor that induces delirium [24]. Hence, we propose that multimodal and multidrug combination analgesia may be beneficial in reducing POD.

### 4.3 Susceptibility of Hyper‐POD in Elderly Patients

In this study, the average age of 7250 patients was 54.5 ± 13 years, and the male/female ratio was approximately 1:1. POD occurs due to multiple factors, among which the complex interaction between susceptible individuals and harmful exposure factors plays a crucial role [19]. Patients were relatively older. Studies have shown that the incidence of POD in elderly patients, especially those over 65 years of age, is 16.85%, which is three times that in patients under 65 years old [25]. This might be due to a decline in the immune capacity and organ compensatory ability with increasing age, resulting in a decreased ability of the body to adapt to external stimuli [26]. Moreover, in elderly patients with low vascular elasticity, intraoperative hypotension can easily lead to insufficient cerebral blood supply and brain tissue hypoxia [27], significantly increasing the probability of POD. Additionally, various medical comorbidities such as cardiovascular diseases, diabetes, lung diseases, arthritis, etc. surgery, and anesthesia, as well as age‐related neurobiological changes (such as reduced brain volume and mass and decreased production of brain neurotransmitters) may lead to more severe complications and longer recovery periods in elderly patients [28]. Therefore, for elderly patients with multiple comorbidities and limited physiological reserves, even relatively mild surgical injuries may induce POD [29, 30].

### 4.4 The Dose of Dexmedetomidine Affects the Incidence of Hyper‐POD

Our study indicates that an appropriate increase in the dexmedetomidine dose can reduce the incidence of postoperative hyperactive delirium. Studies [31] have shown that dexmedetomidine can alleviate the perioperative stress response and inflammatory stimulation in patients and reduce the dosage of analgesic drugs. Furthermore, dexmedetomidine has a relatively small impact on the central cholinergic system. Therefore, dexmedetomidine sedation can reduce the incidence of POD and contribute to the recovery of postoperative cognitive function. Dexmedetomidine is widely used for clinical anesthesia. It protects multiple organs by inhibiting the inflammatory response and activating the anti‐apoptotic signaling pathway, and its use during the perioperative period can significantly reduce the incidence of POD [32, 33, 34]. The results of this study suggest that patients undergoing spinal surgery who use benzodiazepines have a higher risk of POD. Benzodiazepines are commonly used sedatives in anesthesiology; however, they have been identified as high‐risk drugs that induce delirium. The guidelines released by the Society of Critical Care Medicine of the United States in 2018 recommend strategies such as daily interruption of sedation, reduction in sedation, or the use of atypical antipsychotic drugs, dexmedetomidine, and remifentanil as alternatives [30].

### 4.5 The Duration of Surgery Increases the Incidence of Hyper‐POD

Our research shows that prolonged surgery time also increases the incidence of postoperative hyperactive delirium. Prolonged anesthesia time can be understood as a manifestation of longer surgery time and is a known risk factor for POD. Prolonged surgery time may indicate a more complex surgery, increased exposure to microemboli and systemic inflammatory responses, increased postoperative recovery time, and increased risk of complications, which may reduce the ability of patients to regulate neuroinflammation and hypoxia recovery [35]. Long surgeries can also lead to the cumulative aggravation of physiological stress responses, disturbances in blood circulation and metabolism, cumulative effects of anesthetic drugs, sleep deprivation, and other conditions, further increasing the incidence of postoperative hyperactive delirium [36]. We believe that the reduction in surgical time is related to the widespread use of new equipment such as piezosurgery introduced by hospitals. Studies have shown that the piezosurgery device provides a clear operation site because it maintains a blood‐free surgical field during bone cutting, this being attributable to the air‐water cavitation effect of the ultrasonic device. Thus, there should be less intraoperative blood loss and the decompression process should proceed smoothly and take less time [32]. Therefore, for elderly patients, a good preoperative plan and precise and rapid spinal surgery techniques to effectively shorten surgery time are important for reducing postoperative hyperactive delirium. We also believe that during the clinical treatment process, the mental status of patients should be constantly monitored, delirium risk assessment should be conducted, and preventive and intervention measures should be actively implemented [37, 38].

### 4.6 Limitations

This study is a single‐center retrospective study, and there is a possibility of limited sample size and bias. In view of this, large‐scale multicenter retrospective and prospective studies will be the next goal of our research group. We will strive to expand the scope of research, improve the quality of research, and provide more valuable research results for related fields.

## 5 Conclusion

The reduced use of dexmedetomidine, increased use of benzodiazepines, and prolonged surgical time are the reasons for the increased incidence of hyper‐POD. Therefore, to reduce the incidence of hyper‐POD after surgery for degenerative lumbar spine disease, spinal surgeons should perform sufficient preoperative planning and precise surgical steps to shorten the surgical time. At the same time, anesthesiologists can appropriately adjust the proportion of dexmedetomidine and benzodiazepines used during anesthesia. The joint management of orthopedic surgeons and anesthesiologists during the perioperative period is of great significance in reducing the incidence of hyper‐POD in patients undergoing lumbar spine surgery.

## Author Contributions

Dong Yang designed the experiments. Bang‐Lin Xie, Li‐Zhong Nie, Miao Nie, Biao Zhong, and Jun Xiong collected the clinical data. Bang‐Lin Xie and Qiu‐Xiao Ai performed data analysis. Bang‐Lin Xie and Li‐Zhong Nie wrote the article. All authors have read and approved the final article.

## Consent

All authors approved the final article and the submission to this journal. Our research was obtained from all individual participants included in the study.

## Conflicts of Interest

The authors declare no conflicts of interest.

## Data Availability

All data generated or analyzed during this study are included in this published article.
